# Rescuing cellular function in Fuchs endothelial corneal dystrophy by healthy exogenous mitochondrial internalization

**DOI:** 10.1038/s41598-023-30383-8

**Published:** 2023-02-28

**Authors:** Sébastien Méthot, Stéphanie Proulx, Isabelle Brunette, Patrick J. Rochette

**Affiliations:** 1 Axe Médecine Régénératrice, Hôpital du Saint-Sacrement, Centre de Recherche du CHU de Québec – Université Laval, Bureau H2-10, 1050 Chemin Sainte-Foy, Quebec, QC G1S 4L8 Canada; 2 Centre de Recherche en Organogénèse Expérimentale de l’Université Laval/LOEX, Quebec, Canada; 3 Département d’Ophtalmologie, Faculté de Médecine, Université Laval, Quebec, Canada; 4 Maisonneuve-Rosemont Hospital Research Center, Montreal, QC Canada; 5 Ophthalmology Department, Université de Montréal, Montreal, QC Canada

**Keywords:** Mitochondria, Corneal diseases

## Abstract

Fuchs endothelial corneal dystrophy (FECD) is characterized by an accelerated loss of corneal endothelial cells. Since the function of these cells is to maintain the cornea in a state of deturgescence necessary for its transparency, the depletion of corneal endothelial cells ultimately causes corneal edema and irreversible loss of vision. Evidence is accumulating regarding the central involvement of mitochondria in FECD. As we have previously shown, when endothelial cells die and are not replaced, the mitochondria of surviving cells must provide more energy to compensate, leading to a phenomenon we have called mitochondrial burnout. This burnout causes cell death, thus exacerbating an irreversible vicious circle responsible for FECD progression. Corneal transplantation, for which the transplant supply is insufficient, is the only curative alternative for FECD. It thus becomes imperative to find other avenues of treatment. In this article, we tested whether incorporating healthy mitochondria into FECD cells would improve pathological molecular markers of the disease. Using corneal endothelium explants from FECD patients, we demonstrated that incorporation of exogenous mitochondria into FECD cells by co-incubation reduces oxidative stress, increases mitochondrial membrane potential, and reduces mitophagy. In addition, internalization of exogenous mitochondria significantly reduces apoptosis (57% in FECD vs 12% in FECD with internalized mitochondria). Taken together, these results suggest that the internalization of exogenous mitochondria reverses the vicious circle involved in FECD, thus revealing a much-needed novel treatment alternative for FECD.

## Introduction

The cornea is composed of three cellular layers: the epithelium, the stroma and the endothelium. To maintain its transparency, the corneal stroma needs to be partially dehydrated to ensure proper collagen fibrils arrangement^1^. The endothelium is responsible for the maintenance of this partially dehydrated state by pumping ions from the stroma to the anterior chamber using Na^+^/K^+^ ATPase pump^1,2^. Fuchs endothelial corneal dystrophy (FECD) is a corneal pathology characterized by an accelerated loss of endothelial cells and abnormal extracellular deposition (guttae)^3^. When endothelial cell loss reaches a point where the endothelium can no longer perform its role, corneal edema develops, causing vision loss^3^. FECD affects 4 percent of the US population^4^ and the only curative treatment available is corneal transplantation^5,6^. FECD is the first indication for corneal transplantation worldwide, representing about 39% of all corneal transplantations^7^.

The exact cause of FECD is yet to be determined but evidence has shown that oxidative stress and mitochondrial dysfunction are implicated^8,9^. In a previous study, we have shown that oxidative stress and mitochondrial dysfunction are linked in a mitochondrial overuse vicious cycle we called mitochondrial burnout cycle^10^. In this cycle, cells are progressing through different stages that can be sorted using mitochondrial mass. At first, the oxidative stress becomes almost ubiquitous in FECD cells, which damages the mitochondria and leads to the loss of mitochondrial potential (ΔΨ). Some cells will recycle the damaged mitochondria and if the damage is extensive some cells will trigger apoptosis signal. The cell depletion caused by apoptosis forces the remaining cells to increase their mitochondrial activity to pump more ions per cell. To respond to the increased energetic demand, these cells increase their mitochondrial mass, which leads to an increased production of reactive oxygen species (ROS). These ROS further damage the cells that will eventually trigger mitophagy and apoptosis^10^.

In agreement with the data showing that mitochondria play a central role in FECD pathogenesis^8,10,11^, we hypothesized that the incorporation of functional mitochondria in FECD-affected endothelial cells would reverse some cellular phenotypes of the disease. It was previously shown that isolated mitochondria could be internalized by cells via macropinocytosis^12^. We hypothesized that the internalization of mitochondria in FECD cells could be exploited as a treatment for this pathology^12^. This strategy is already being investigated as a potential treatment for different pathologies linked to mitochondrial dysfunction, such as Parkinson disease or ischemia–reperfusion injury^13,14^. In this study, we incubated corneal endothelial explants from FECD patients with isolated mitochondria from cultured cells, to observe their internalization. We then showed that the internalization of exogenous mitochondria in FECD cells by co-incubation reduces oxidative stress and increases mitochondrial membrane potential (ΔΨ), reversing the mitochondrial burnout markers. Moreover, our results show that the internalization of healthy mitochondria reverses apoptosis in FECD cells. Taken together, our data show that mitochondrial transplantation rehabilitates the corneal endothelial phenotype in FECD explants.

## Materials and methods

All experiments in this study were performed in accordance with the Declaration of Helsinki and the research protocol was approved by the CIUSSS-EMTL (Montréal) and the CHU de Québec-Université Laval (Québec) institutional ethics committees for the protection of human subjects, with patients’ written informed consent for study participation. FECD explants (corneal endothelium and Descemet’s membrane) were obtained from 23 consenting patients with late stage FECD at the time of their corneal transplantation (Centre universitaire d’ophtalmologie (CUO)—Hôpital Maisonneuve-Rosemont (HMR) and CUO—Hôpital du Saint-Sacrement, Québec, Canada). Every cellular portion of each FECD explant was analyzed without discrimination between the central and peripheral portions of the explant. Patients’ age (mean 56–86 years; median 71; SD ± 8.2) and sex are listed in Supplementary Material (Table S1).

### Mitochondria extraction and transplantation by co-incubation

Immortalized human embryonic kidney cells (HEK-293T; American Type Culture Collection, #CRL-3216) were cultured in Dulbecco’s modified Eagle’s Medium (DMEM; Wisent, Canada) supplemented with 5% fetal bovine serum (FBS) (Sigma, Canada) and 1% penicillin/streptomycin (Wisent, Canada) at 37 °C, 8% CO_2_. At full confluency, the cells were harvested using a cell scraper in PBS and resuspended in mitochondria isolation buffer (Mi): 300 mM sucrose, 1 mM EGTA/Tris, 5 mM MOPS/Tris, 5 mM KH_2_PO_4_, adjusted to pH 7.4 with KOH. The suspension was then homogenized using a Teflon pestle in a glass tube and filtered through 40 μm, 10 μm and 5 μm polyethylene terephthalate (PET) filters (pluriSelect, Germany)^15^. The filtrate was centrifugated at 6000×*g* for 10 min and the supernatant was discarded and replaced with fresh Mi. Protein content of the mitochondrial extraction was measured using the protein assay dye (Bio-Rad, USA) based on the Bradford method according to the manufacturer’s protocol. Mitochondria were then diluted at a concentration of 0.2 mg/mL in DMEM 5% FBS 1% penicillin/streptomycin and co-incubated with explants at 37 °C 8% CO_2_.

### Internalization of exogenous mitochondria

To observe mitochondrial cellular internalization, we added pre-stained mitochondria to the culture medium of FECD explants. The mitochondria extracted from HEK-293T cells were stained with 0.1 μL of Mitotracker Green (Invitrogen) 1 mM per mg of mitochondrial protein for 30 min at 37 °C. Stained mitochondria were centrifuged at 6000×*g* for 10 min, the supernatant was discarded and mitochondria were resuspended in 500 µL Mi. Stained mitochondria, at a concentration of 0.2 mg/mL, were then co-incubated with the explants for 3 h, after which explants were stained with 80 mM Mitotracker Deep Red (Invitrogen) in PBS for 30 min at 37 °C. Microscopic examination of the explants was followed by image analysis to measure the content of internalized mitochondria (green) and endogenous mitochondria (red).

### Mitochondrial potential

Explants were cut in 2 equal parts; one half was incubated in DMEM, while the other half was incubated with 0.2 mg/mL of isolated mitochondria in DMEM for 3 h or 48 h. Explants were then stained in a solution of 2.5 μM JC-1 (Invitrogen) and 80 mM Mitotracker Deep Red diluted in PBS for 30 min at 37 °C. Microscopic examination of the explants was followed by image analysis to determine the ratio of red JC-1 on Mitotracker Deep Red.

### Oxidative stress

Explants were cut in 2 equal parts; one half was incubated in DMEM (Control), while the other half was incubated with 0.2 mg/mL of isolated mitochondria in DMEM for 48 h (Mitochondria). Explants were stained in a solution of 2.5 μM CM-H2DCFDA (Invitrogen) and 80 nM Mitotracker Deep Red diluted in PBS for 30 min at 37 °C. Microscopic examination of the explants was followed by image analysis to determine the level of CM-H2DCFDA per cell.

### Mitophagy

Explants were cut in 2 equal parts; one half was incubated in DMEM (Control) and the other half was incubated with 0.2 mg/mL of isolated mitochondria in DMEM for 24 h (Mitochondria). Explants were stained in a solution of 75 nM of Lysotracker Deep Red (Invitrogen) diluted in PBS for 2 h at 37 °C, washed with PBS, and stained with a solution of 80 nM of Mitotracker Green diluted in PBS for 30 min at 37 °C. Microscopic examination of the explants was followed by image analysis to determine the level of Mitotracker Green that colocalized with Lysotracker Deep Red per cell^16^.

### Apoptosis

Explants were cut in 2 equal parts; one half was incubated in DMEM (Control), while the other half was incubated with 0.2 mg/mL of isolated mitochondria in DMEM for 48 h (Mitochondria). Explants were stained in a solution of 5 μM of Caspase-3/7 Green Detection Reagent (Invitrogen) and 80 nM Mitotracker Deep Red diluted in PBS 5% FBS for 30 min at 37 °C. Microscopic examination of the explants was followed by image analysis to determine the ratio of caspase-3/7 positive cells on cell total.

### Image and statistical analysis

The signal produced by the different markers used for internalization, mitochondrial potential, oxidative stress and apoptosis experiments was quantified using AxioVision 4.8.2 (Zeiss, Germany). Analyses were performed as described previously for each experiment^10^. The signal produced by the mitophagy experiment was measured using Image J (NIH, USA) with the EzColocalisation plugin. Analysis was performed as previously described^17^.

To generate statistical analysis, Kaleida graph version 4.1.3 software (Synergie software, Readint, PA, USA) was used. Differences between Control and Mitochondria treated explants were assessed with the paired two-tailed homoscedastic Student’s t-test. A p-value ≤ 0.05 was defined as statistically significant.

## Results

### Mitochondrial internalization efficiency correlates with initial mitochondrial mass in FECD cells

**Figure 1:**
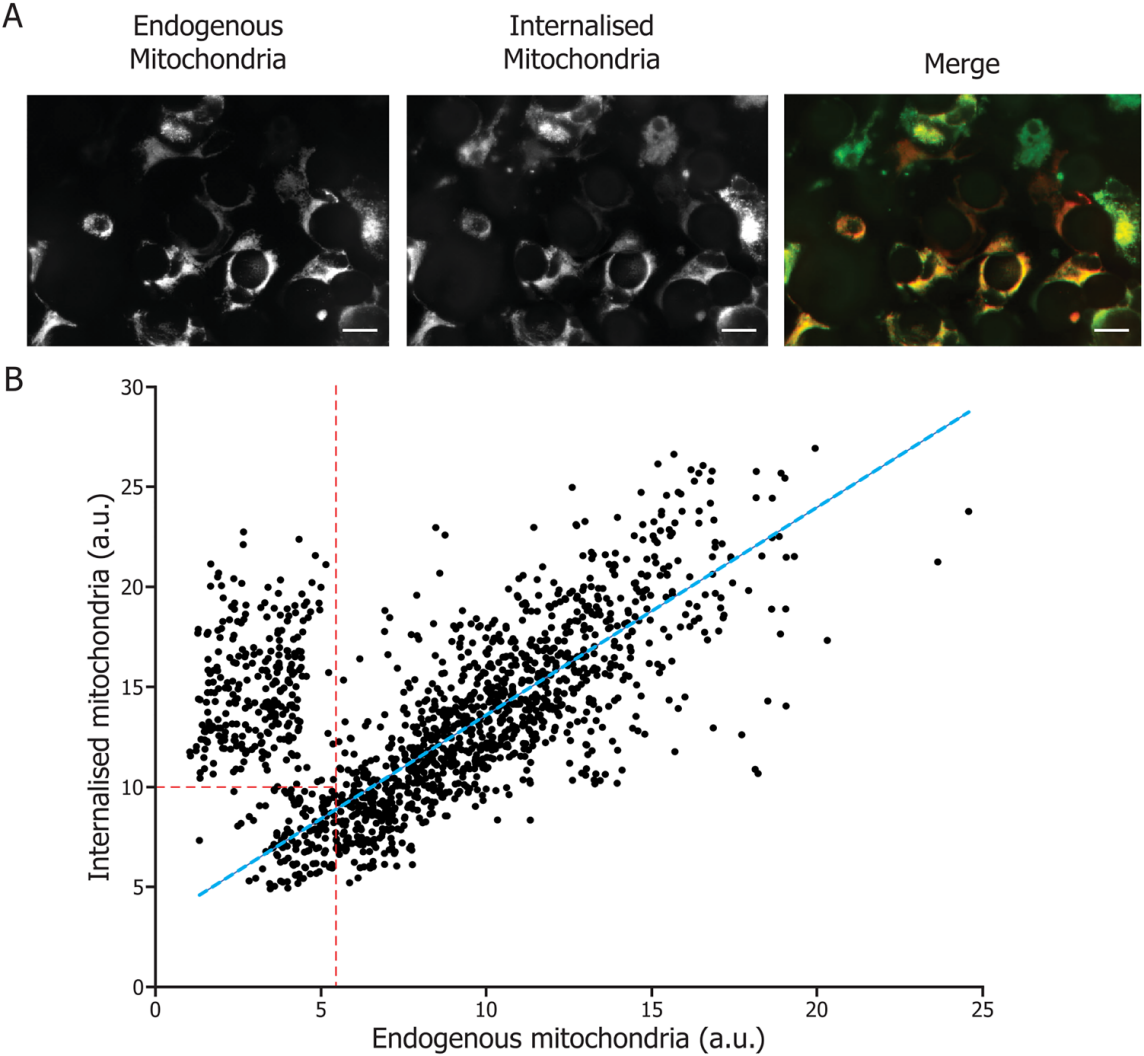
Endogenous mitochondrial mass in FECD cells influences mitochondrial internalization. (**A**) Mitochondrial mass markers (mitotracker) were used to label exogenous internalized (green) and endogenous mitochondria (red). (**B**) Both mitotracker signals were plotted, each dot representing a single cell. The mitochondrial mass appeared to be distributed according to two distinct clusters that could be arbitrarily separated by the two red dotted lines drawn in Figure, the horizontal red dotted line at 10 a.u. illustrating the internalized mitochondria threshold and the vertical red dotted line representing the 27% lowest endogenous mitochondrial mass level (5.2 a.u.). A strong positive correlation is found between the amounts of endogenous and exogenous mitochondria in the cells outside the red dotted line enclosed area (R2 = 0.66; ρ = 0.66; p < 0.001). Scale bar = 40 µm. Experiments were performed with 3 explants from 3 FECD patients (1555 cells analyzed).

Pre-labelled mitochondria were co-incubated with corneal endothelial explants from a FECD patient for 3 h. The explant was then labelled with a different mitochondrial marker to assess endogenous mitochondrial mass. We observed an important variation in incorporation efficiency among endothelial cells (Fig. 1A), which was confirmed by the quantification of the Mitotracker signal in individual cells (Fig. 1B). A positive correlation was observed between internalized and endogenous mitochondrial mass values, apart from a portion of the cells with the lowest level of endogenous mitochondria. These cells represent 18% of all cells. The correlation between the internalized and initial mitochondrial mass values in the remaining 82% of the cells was found to be strongly positive (R2 = 0.66; ρ = 0.81; p < 0.0001) (Fig. 1B). The internalization of mitochondria thus appeared to be linked to the level of endogenous mitochondria, the cells with the highest level of endogenous mitochondria internalizing the most exogenous mitochondria.

### Mitochondrial internalization in FECD explants by co-indubation leads to an increase of mitochondrial membrane potential

**Figure 2:**
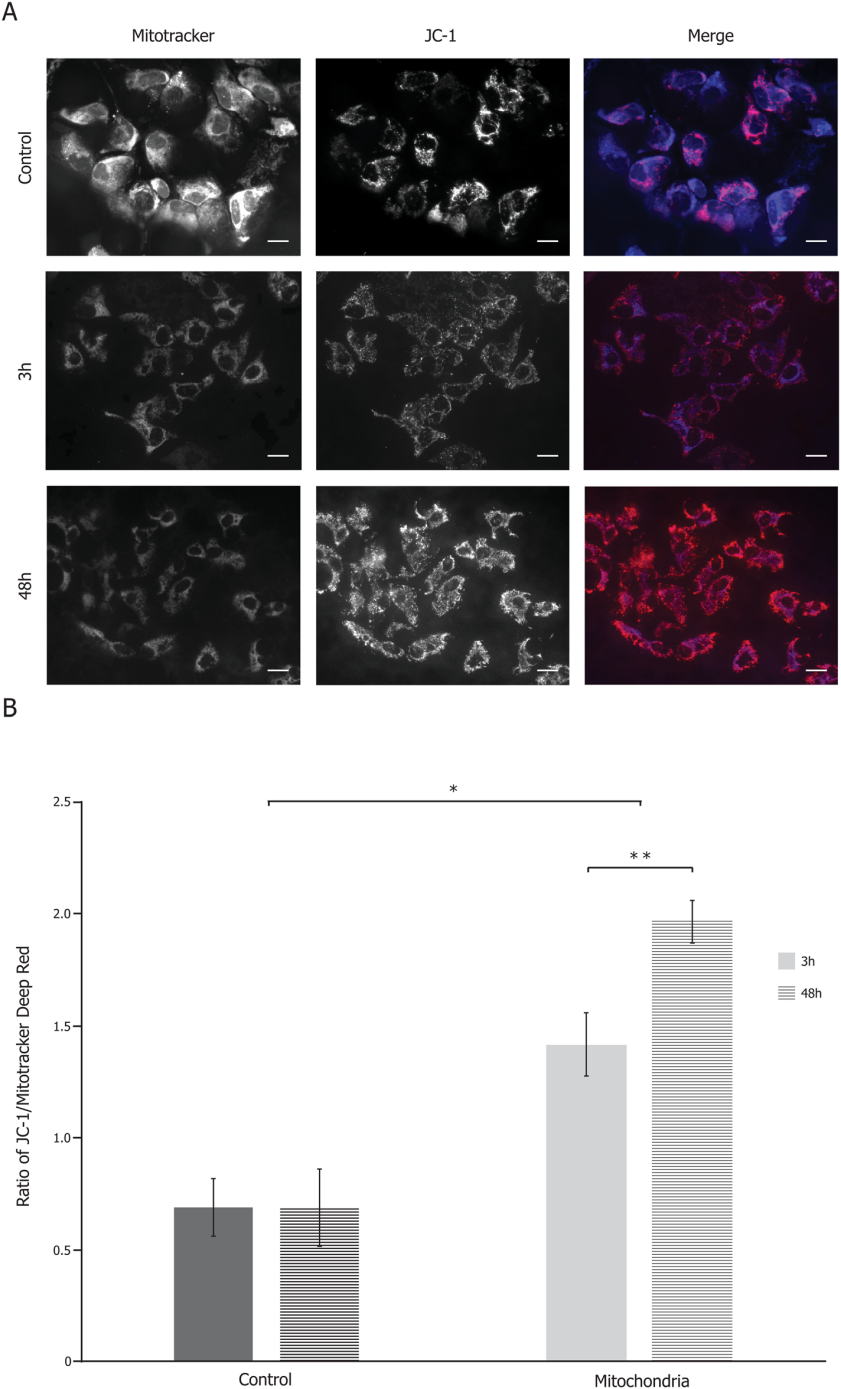
Mitochondria internalization improves mitochondrial membrane potential (ΔΨ) in FECD cells. (**A**) A marker of ΔΨ (JC-1, red) was used in conjunction with a mitochondrial mass marker (mitotracker; blue) for all FECD explants of this experiment, exposed to exogenous mitochondria or not (Control) for 3 h or 48 h. (**B**) Ratio of ΔΨ/mitochondrial mass (JC-1 on mitotracker) is higher in the explants exposed to exogenous mitochondria than in the controls. This increase is statistically significantly more pronounced at 48 h than at 3 h. Scale bar = 40 µm. Serial measurements at each time point were performed on 4 explants from 4 FECD patients (47 microscope fields analyzed at 3 h; 41 fields at 48 h). *p < 0.005; **p < 0.001.

FECD explants were cut in 2 equal parts, one of them was incubated with exogenous mitochondria (Mitochondria), while no mitochondria were added to the medium of the other one (Control). After 3 h or 48 h of incubation, the mitochondrial membrane potential (ΔΨ) was measured. We observed an increase of ΔΨ when cells were treated with fresh mitochondria (Fig. 2A). The ΔΨ signal and the mitochondrial mass were measured to derive the ratio of ΔΨ per mitochondria (JC-1/Mitotracker) (Fig. 2B). The ratio was significantly higher in the mitochondria-treated endothelia (1.42 for 3 h and 1.97 for 48 h) compared to untreated controls (0.69 for 3 h and 48 h). The ratio increased with time and was significantly higher at 48 h (1.97) than at 3 h (1.42).

### Oxidative stress in FECD explants is decreased upon exogenous mitochondrial co-incubation

**Figure 3:**
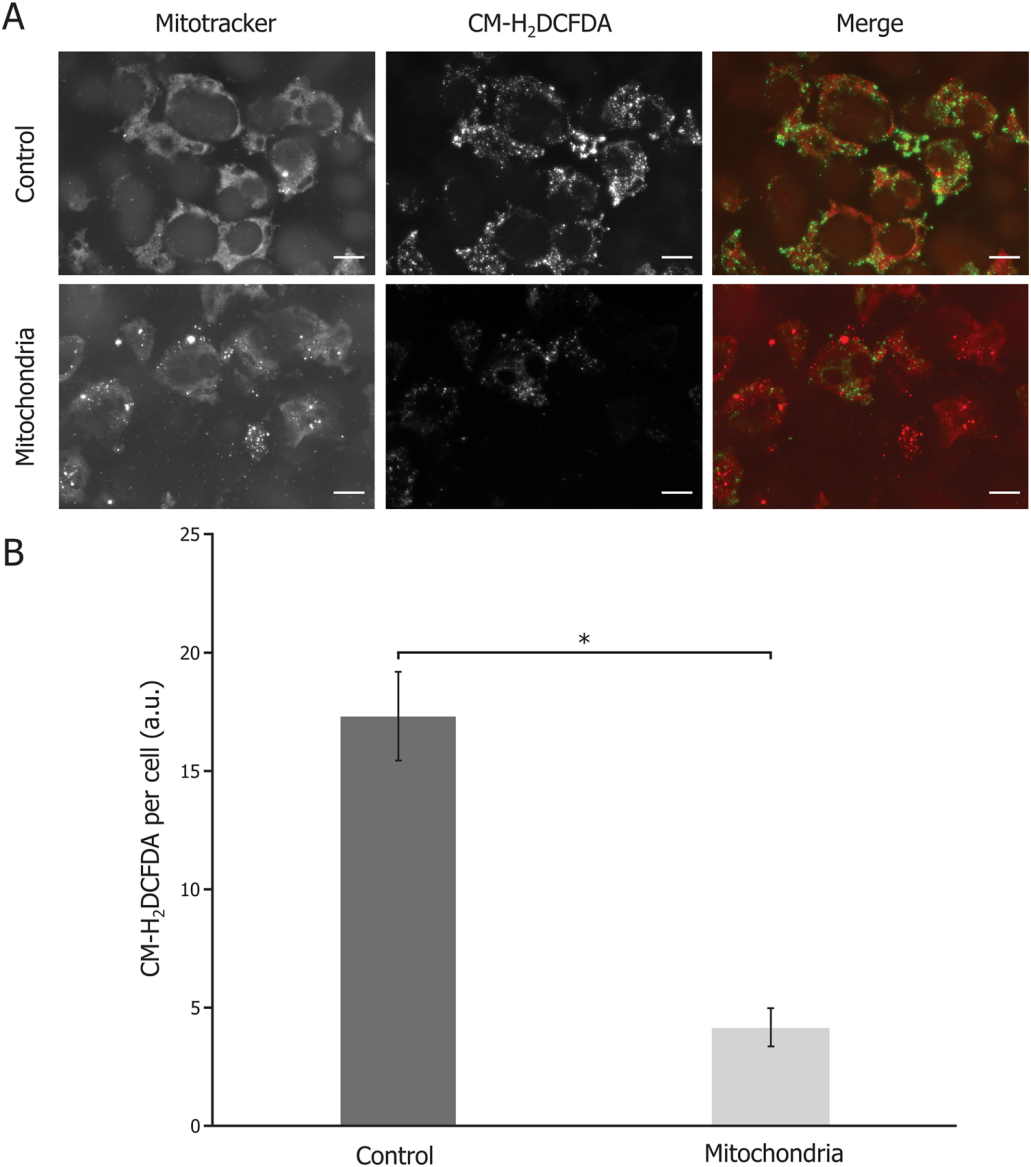
Oxidative stress is reduced in FECD cells when exposed to exogenous mitochondria. (**A**) A marker of oxidative stress (CM-H2DCFDA; green) was used in conjunction with a mitochondrial mass marker (mitotracker; red) in FECD explants exposed to exogenous mitochondria or not (Control). (**B**) CM-H2DCFDA signal per cell is significantly reduced in FECD explants that received exogenous mitochondria, compared to control explants. Scale bar = 40 µm. Experiments were performed on 4 explants from 4 FECD patients (Total 41 microscope fields analyzed). *p < 0.005.

We measured the oxidative stress level using FECD explants cut in two equal parts, one exposed to exogenous mitochondria for 48 h while the other was used as a control. Less accumulation of ROS was observed in the mitochondrial-treated portions of the FECD explants (Fig. 3A), the signal quantification of CM-H2DCFDA per cell being significantly lower in mitochondria-treated endothelia (4.2 a.u.) than in the controls (17.3 a.u.) (Fig. 3B).

### Mitophagy in FECD cells is reduced after exogenous mitochondria co-incubation

**Figure 4:**
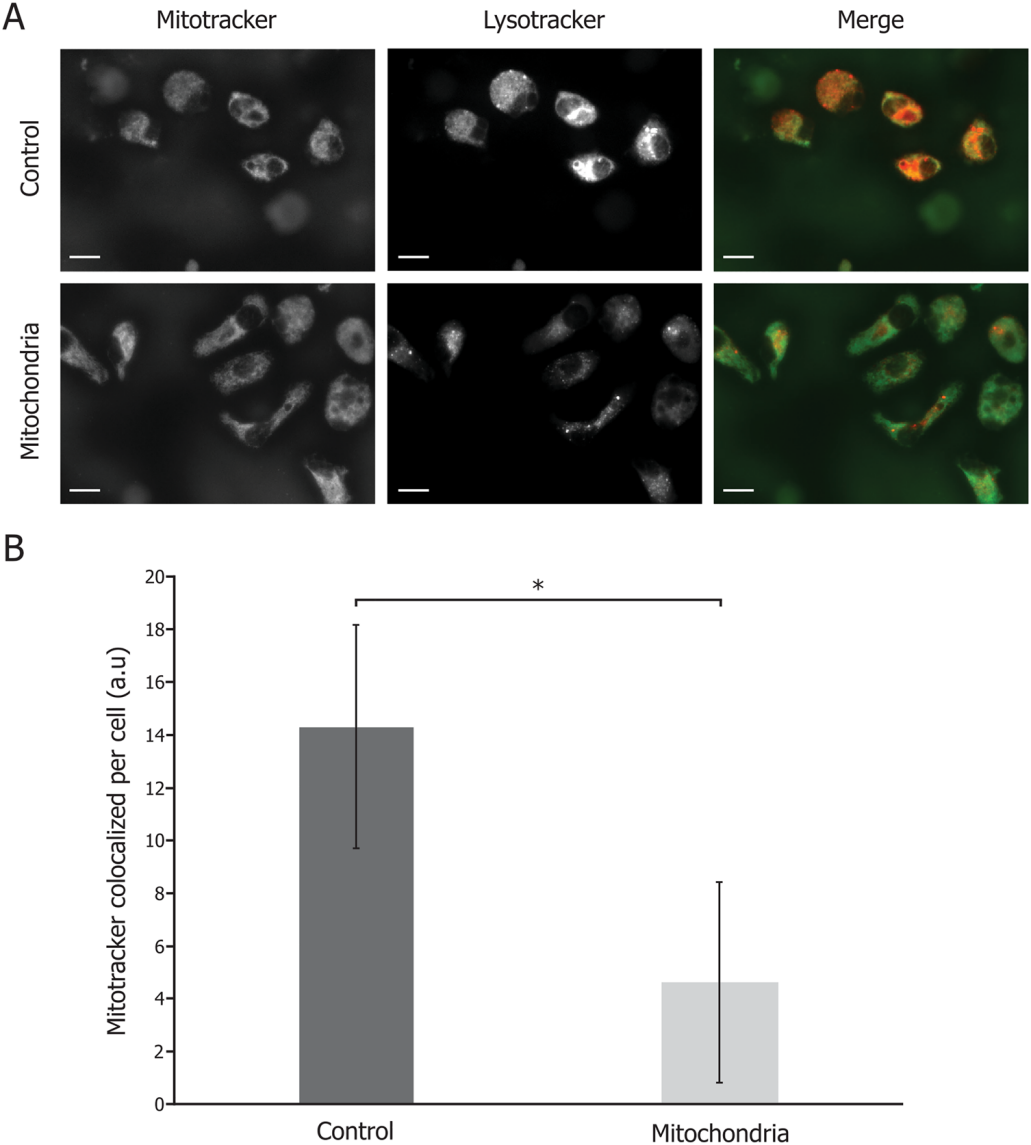
Reduction of mitophagy by mitochondria internalization by co-incubation with FECD cells. (**A**) A marker of lysosome (lysotracker; red) was used in conjunction with a mitochondrial mass marker (mitotracker; green) in FECD explants exposed to exogenous mitochondria or not (Control). (**B**) Colocalization of the mitotracker and lysotracker signals is lower in the FECD explants that received exogenous mitochondria compared to controls. Scale bar = 40 µm. Experiments were performed with 4 explants from 4 FECD patients (43 microscope fields analyzed). *p < 0.005.

Mitophagy was measured in FECD using explants cut in two equal parts, one treated with mitochondria for 2 hs and the other used as an untreated control. A lower level of mitophagy was observed in FECD explants treated with exogenous mitochondria (Fig. 4A) and the signal of Mitotracker colocalized with Lysotracker was significantly lower in mitochondria-treated explants (14.0 a.u.) compared to Control explants (4.6 a.u.) (Fig. 4B).

### Apoptosis in FECD is reduced by exogenous mitochondrial co-incubation

**Figure 5:**
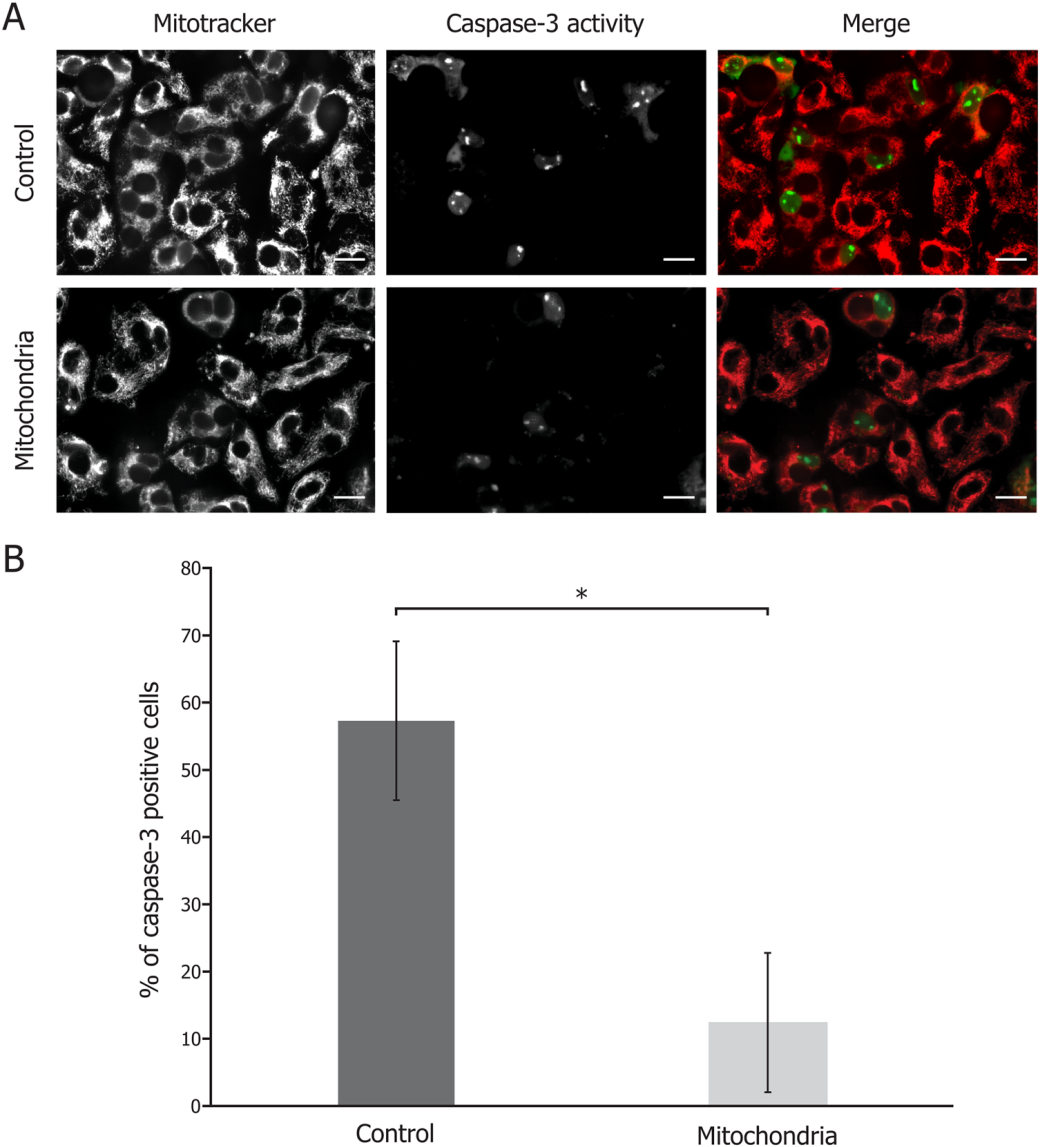
Apoptosis is reversed by exogenous mitochondria internalization by co-incubation with FECD cells. (**A**) A marker of apoptosis (caspase 3/7 activity; green) in conjunction with a mitochondrial mass marker (mitotracker; red) in FECD explants that received exogenous mitochondria or not (control). (**B**) The number of apoptotic cells is lower in mitochondrial treated FECD explants, when compared to control. Scale bar = 40 µm. Experiments were performed with 4 explants from 4 FECD patients (38 microscope fields analyzed). Other representative images of the 4 explants have been added to the Supplementary Materials (Fig. S3). *p < 0.001.

We measured apoptosis in FECD using explants cut in two equal parts, one of which treated with mitochondria for 48 h and the other used as untreated control. We measured fewer apoptotic cells in FECD treated with mitochondria halves (Fig. 5A). Indeed, the percentage caspase-3/7 was significantly lower in FECD explants treated with mitochondria halves (12.4%) when compared to the controls (57.3%) (Fig. 5B).

## Discussion

In this study, we used FECD explants obtained at the late stage of the disease. We used FECD explants instead of cultured FECD corneal endothelial cells since we and other have shown that the culture process results in a cell phenotype closer to non-pathological cells, in regards to pump and barrier functionality^18,19^, either by selecting the cells that are the least advanced in the pathology, or by removing the cells from their underlying diseased environment (aqueous humor, Descemet membrane). Thus, to our knowledge, explants remain the best model to adequately study FECD. The main limitation of this model is the quantity of cells in the explant, which prevents us from performing more traditional molecular and cellular biology analyzes (e.g., Western blot, flow cytometry). Even if the explants come from patients at an advanced stage of the disease, we have shown, using the same techniques as the one employed in this article, that in the same explant, individual cells are at different stages of the pathology^10^. We have also shown that endothelial mitochondrial mass can be used as a cellular marker of different stages of progression of FECD^10^.

In this article, we show that mitochondrial incorporation efficiency depends on the initial level of mitochondrial mass (Fig. 1), suggesting that the stage of progression of the disease affects the efficiency by which mitochondria enter the cells. In this study, incorporation of exogenous mitochondria seemed to be higher in cells with either the lowest or the highest levels of endogenous mitochondria. In almost all control cells (untreated with mitochondria) in FECD explants had a reduced ΔΨ, most likely due to damage caused by ROS^10^. The introduction of exogenous mitochondria caused an increase of ΔΨ as soon as 3 h after exposure (Fig. 2). After 48 h, ΔΨ was still rising most likely due to the removal of damaged mitochondria via mitophagy and the persistent presence of the fully functional mitochondria introduced. Indeed, it has been shown that when healthy mitochondria are incorporated into cells containing damaged mitochondria, these damaged mitochondria are being replaced by healthy ones^13,20,21^. The sustained increase in ΔΨ following mitochondrial transplantation suggests that this treatment could restore the ATP production that was lost in FECD cells^22^.

FECD cells containing the highest levels of endogenous mitochondria were previously shown to also face a higher oxidative stress, although they were not the ones undergoing apoptosis^10^. These cells are most likely trying to compensate for the cell loss caused by FECD progression by increasing ATP production, which leads to an increase in ROS production. In agreement with our results depicted in Fig. 3 showing a reduction of ROS in FECD explants treated with mitochondria, it has previously been shown that mitochondrial transplantation reduces oxidative stress and ROS production in other tissues/cells^23,24^. This effect is most likely attributed to the antioxidant defenses brought to the cell by the exogenous mitochondria. Our team and others have shown that FECD cells harbor mtDNA deletions caused by oxidative stress^9,18^. Cumulative mtDNA damage leads to an increase in ROS production, forcing cells to eliminate mitochondria with damaged mtDNA via mitophagy, a process further generating ROS^25,26^. Increased levels of mitophagy were documented in FECD cells^22,27^. Taken together, these data suggest that FECD cells are in a constant flux between mitophagy to remove damaged mitochondria and ROS production by the damaged mitochondria, which in turn produces more damaged mitochondria. This vicious cycle could theoretically be broken by the internalization of healthy mitochondria producing ATP without generating aberrant ROS, allowing cells to discard their damaged mitochondria and still produce enough energy to insure their function. This would explain the reduced level of mitophagy observed following mitochondrial internalization (Fig. 4). Mitochondrial transplantation could thus prevent the resisting cells from progressing further within the FECD cycle and prevent them from undergoing apoptosis.

In a previous study, we showed that FECD endothelial cells with the lowest mitochondrial mass had a ΔΨ almost absent and were undergoing apoptosis^10^. Here we show that internalization of exogenous mitochondrial leads to a reduction of the number of apoptotic cells in FECD explants (Fig. 5). Caspases activation is usually seen as a point of no return for the apoptotic cells^28,29^, however recent studies have challenged this dogma^30^. Anastasis refers to the different mechanisms enabling cells to bypass death and return from programmed cell death under certain conditions, even when at very late stage of the process^30–32^. One of these conditions is energy availability^30^. During apoptosis, mitochondria are impaired by the opening of the apoptotic pores resulting in the release of cytochrome c. This leaves the cells without sufficient energy production to reverse apoptosis. However, this condition can be artificially reversed by the internalization of exogenous mitochondria, as evidenced by the subsequent increase in ΔΨ that restores energy production of the cell (Fig. 2). Moreover, the exogenous mitochondria alleviate the oxidative stress, an important apoptosis trigger (Fig. 3). Altogether, mitochondrial transplantation seems to gather the conditions for anastasis to occur in FECD cells, thus preventing cell loss and potentially delaying progression of the disease.

Our observations showed that mitochondrial internalization improves the FECD pathological phenotype at the molecular and cellular levels^8,11,33^. It reduces the oxidative stress status, it reverses apoptosis and brings back FECD corneal endothelial cells to life. This reveals an entirely new avenue for the treatment of FECD. Our analyzes were carried out over a period of up to 48 h. Maintaining explants in culture over a longer period is difficult to envisage. Studies on in vivo models will have to be conducted to confirm the long-term effect of mitochondrial incorporation. However, it is obvious that such a substantial improvement in such a short period of time is extremely promising for the future. Any type of rehabilitation of the FECD endothelium allowing to avoid or delay corneal decompensation and corneal transplantation will constitute an unprecedented advance in the management of this blinding disease. We also believe that this highly innovative concept may find promising potential applications in other eye diseases for which mitochondrial dysfunction has been reported to be central.

## Supplementary Information

Supplementary Information.

## Data Availability

All data generated or analysed during this study are included in this published article and its Supplementary Information files.
