# Supplementary material for: Rescuing cellular function in Fuchs endothelial corneal dystrophy by healthy exogenous mitochondrial internalization
Source: Sci Rep. 2023 Feb 28;13:3380. doi: 10.1038/s41598-023-30383-8 (PMC9974992; doi:10.1038/s41598-023-30383-8)
Supplement: Supplementary file 1 — Supplementary Information. [file 41598_2023_30383_MOESM1_ESM.pdf]

Supplementary material

Rescuing cellular function in Fuchs endothelial corneal dystrophy by healthy exogenous mitochondrial internalization

Sébastien Méthot[1,2], Stéphanie Proulx[1,2,3], Isabelle Brunette[4,5], Patrick J. Rochette[1,2,3,*]

1. Centre de Recherche du CHU de Québec – Université Laval, Axe Médecine Régénératrice, Hôpital du Saint-Sacrement, Québec, Canada
2. Centre de recherche en organogénèse expérimentale de l'Université Laval/LOEX, Québec, Canada
3. Université Laval, Faculté de Médecine, Département d'Ophtalmologie, Université Laval, Québec, Canada.
4. Maisonneuve-Rosemont Hospital Research Center, Montreal, Québec, Canada
5. Université de Montréal, Ophthalmology Department, Montreal, Québec, Canada

* Corresponding author:
Dr. Patrick J. Rochette
Centre de recherche du CHU de Québec – Université Laval
Axe médecine Régénératrice
Hôpital du Saint-Sacrement, Bureau H2-10
1050 Chemin Sainte-Foy, Québec, Qc, Canada, G1S 4L8
Phone: (418) 682-7568
E-mail: Patrick-J.Rochette@crchudequebec.ulaval.ca

SUPPLEMENTARY FIGURES

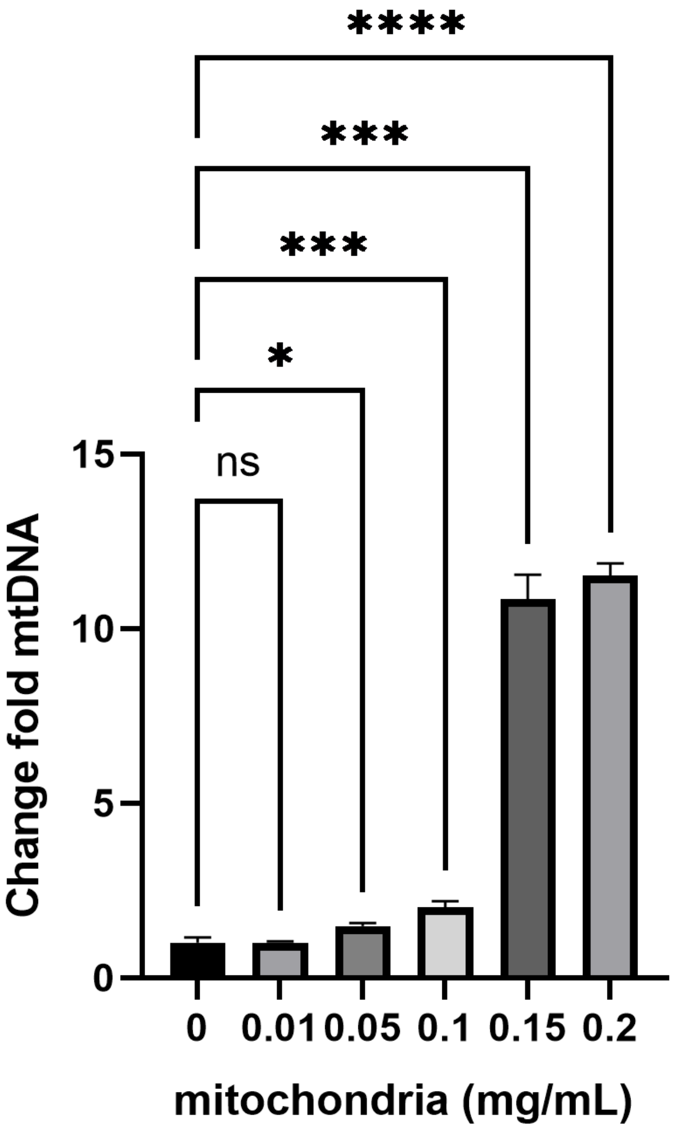

Figure S1. Level of exogenous mitochondrial incorporation in human cells. Different concentration of extracted mitochondria from 293T (0 – 0.2 mg/mL) were co-incubated with human diploid dermal fibroblasts for 3h. Relative mtDNA level was assessed using Q-PCR as we have previously described (Gendron et al, IOVS 2016). Briefly, the amplification level of mtDNA primers was compared with the 18S genomic DNA coding region, and a mtDNA molecules-to-18S coding region ratio (mtDNA:18S DNA) was derived. The level of mtDNA was increased by at least 10x when 0.2 mg/mL of exogenous mitochondria was co-incubated with dermal fibroblasts. Experiment has been performed 3x (N=3) in quadruplicate (n=4).

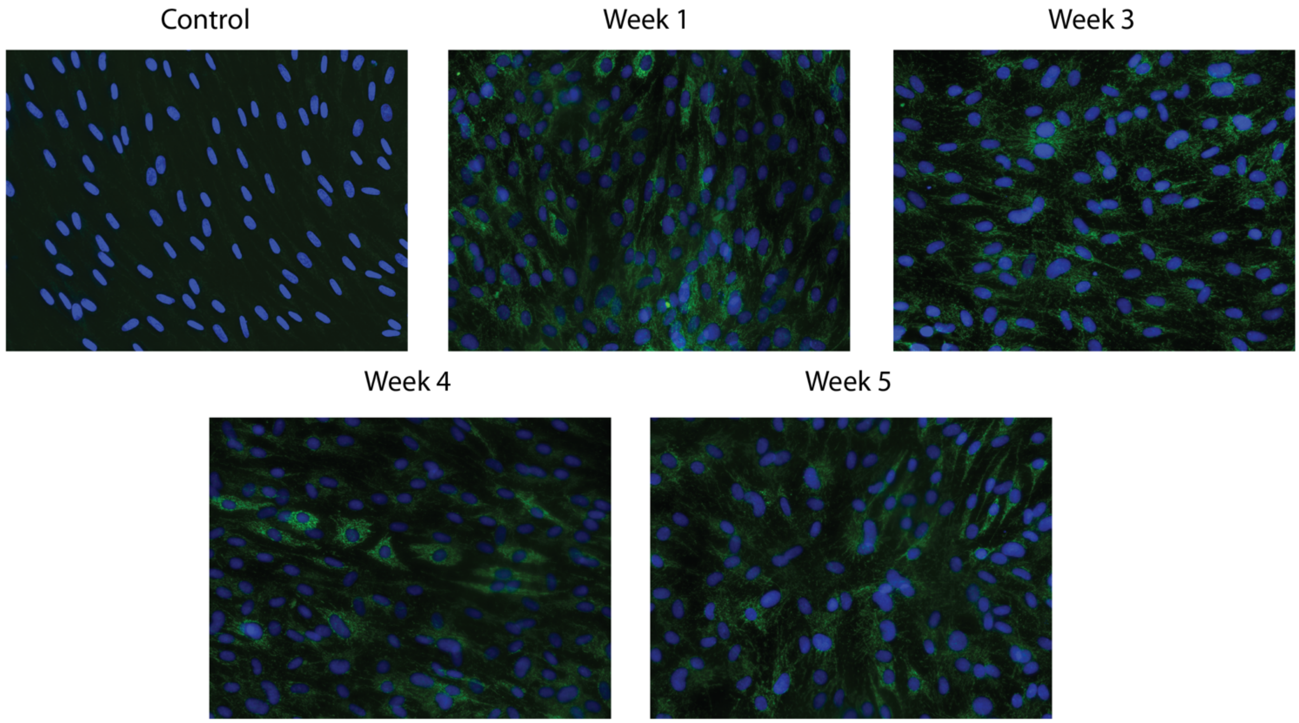

Figure S2. Measure of long-term persistence of exogenous mitochondria in host cells. Corneal rabbit fibroblasts at confluency were co-incubated with 0.2 mg/mL of mitochondria extracted from human cells. After 24h incubation, cells were washed with PBS and fresh culture medium was added. One petri of cell was collected and fixed in methanol after each week for 5 weeks. Anti-mitochondria antibody reacting only with human mitochondria (Abcam, Cat# AB92824) was used (green) according to the manufacturer protocol. Nuclei were counterstained with DAPI (blue). This result confirm that exogenous mitochondria persist in the cells for at least 5 weeks. This experiment has been performed with one cell culture (N=1).

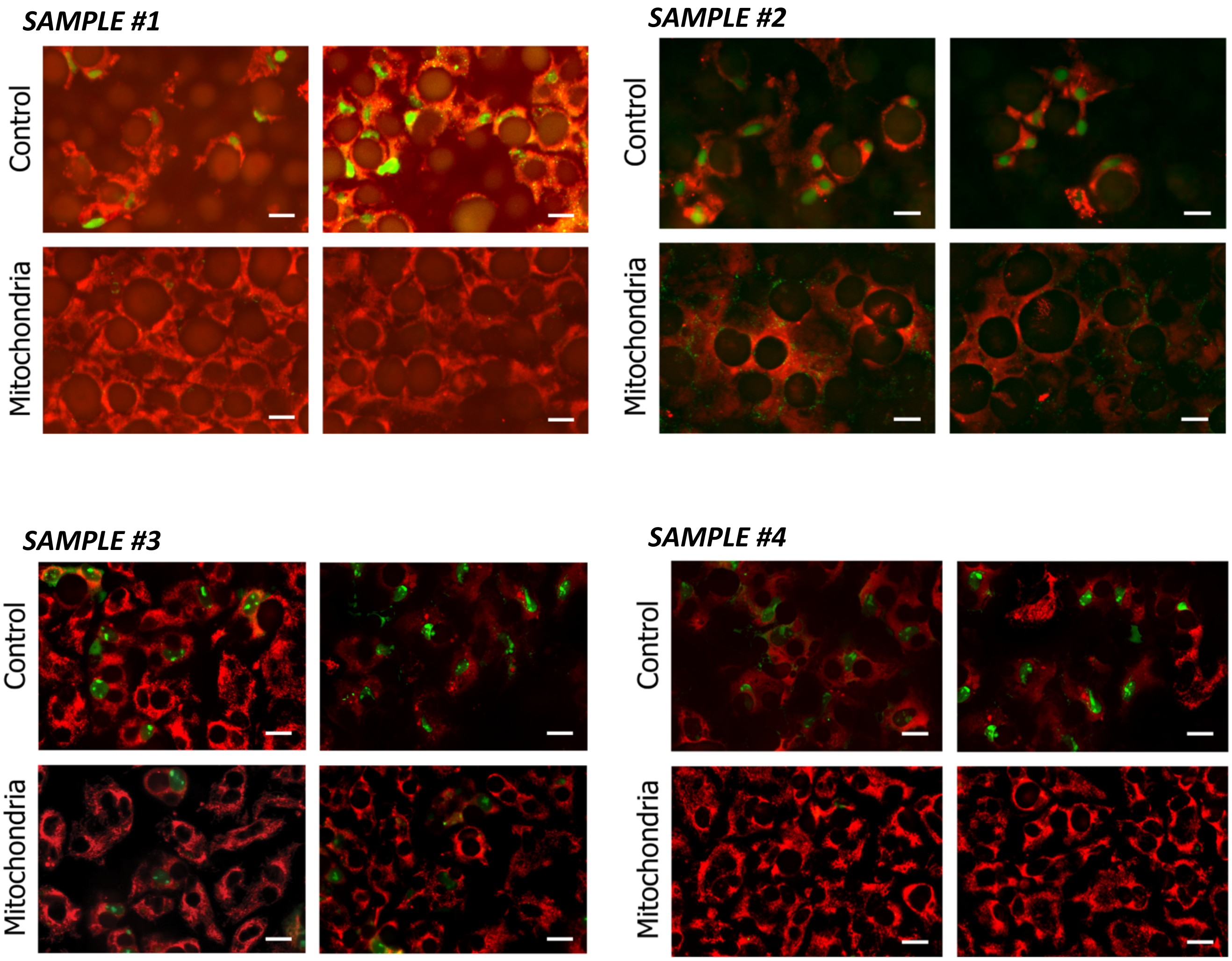

Figure S3. Additional representative images of apoptosis reversion by exogenous mitochondria internalization in FECD cells. Marker of apoptosis (caspase 3/7 activity; green) was used in conjunction with a mitochondrial mass marker (mitotracker; red) in FECD explants that has been co-incubated with 0.2mg/mL of exogenous mitochondria.

SUPPLEMENTARY TABLE

Table S1. List of samples used in the study.

Age	Sex	Figure	Number of Fields	
			Control	Mitochondria
69	Male	1	-	5
71	Female	1	-	5
72	Male	1	-	10
71	Male	2	4	4
74	Male	2	6	6
65	Female	2	7	7
65	Male	2	6	7
74	Female	2	6	6
82	Male	2	5	7
79	Female	2	4	7
86	Male	2	4	5
77	Female	3	4	6
62	Female	3	5	5
66	Female	3	6	5
62	Male	3	5	5
56	Male	4	5	3
79	Female	4	5	6
59	Male	4	6	7
57	Male	4	6	5
60	Female	5	6	5
74	Male	5	4	4
72	Female	5	5	5
63	Female	5	4	5
